# Allogeneic Stem Cell Transplantation in Patients With Mixed Chimerism Following Double Cord Blood Transplantation: A Case Report

**DOI:** 10.1155/crh/2052096

**Published:** 2026-06-12

**Authors:** Youngrok Park, Seonghan Lee, Jinhang Kim, Jeong-A Kim

**Affiliations:** ^1^ Tumor Biology Training Program, Lombardi Comprehensive Cancer Center, Georgetown University School of Medicine, Washington, DC, USA, georgetown.edu; ^2^ Department of Hematology, College of Medicine, Seoul St. Mary’s Hospital, Catholic Hematology Hospital and Leukemia Research Institute, The Catholic University of Korea, Seoul, South Korea, medcol.mw; ^3^ Department of Internal Medicine, Division of Hematology, College of Medicine, St. Vincent Hospital, The Catholic University of Korea, Seoul, South Korea, medcol.mw

**Keywords:** allogeneic peripheral stem cell transplantation, case report, cord blood transplantation, donor selection, mixed donor chimerism

## Abstract

**Introduction:**

Double‐unit cord blood transplantation (dCBT) is an established strategy to enhance progenitor cell dose and accelerate hematopoietic recovery when a matched sibling or unrelated donor is unavailable. While dCBT typically results in the dominance of a single unit, the persistence of donor–donor mixed chimerism is an exceptionally rare clinical phenomenon. This case report describes a unique therapeutic strategy where persistent donor–donor chimerism following dCBT was utilized to redefine the recipient’s HLA profile for a subsequent successful allogeneic transplantation.

**Case Presentation:**

A 38‐year‐old patient with diffuse large B‐cell lymphoma experienced disease relapse following autologous peripheral blood stem cell transplantation. After achieving a second remission through salvage chemotherapy, the patient underwent dCBT due to the absence of a compatible HLA‐matched donor. Fifteen months posttransplantation, the disease relapsed again; however, further salvage therapy successfully induced a third remission. Notably, HLA analysis revealed persistent donor–donor mixed chimerism with one unit maintaining clear dominance. Because no suitable matches existed for the patient’s native HLA type, the dominant donor’s HLA genotype was utilized as the new target for donor selection. This shift facilitated the identification of a matched allogeneic peripheral blood stem cell transplantation (allo‐PBSCT) donor.

**Conclusion:**

The patient subsequently underwent a second allo‐PBSCT based on the dominant donor’s HLA profile. The procedure resulted in robust engraftment and complete donor chimerism without the occurrence of graft‐versus‐host disease. The patient has remained in relapse‐free survival for over 4 years. This case demonstrates that in the rare event of persistent donor–donor chimerism post‐dCBT, the emergent dominant HLA genotype can serve as a viable and effective target for donor selection, providing a life‐saving alternative for patients who lack native HLA‐matched donors.

## 1. Introduction

Allogeneic peripheral blood stem cell transplantation (allo‐PBSCT) is a definitive curative option for hematologic malignancies, yet its success hinges on human leukocyte antigen (HLA) compatibility to prevent graft failure and graft‐versus‐host disease (GVHD). For the nearly 50% of patients without an 8/8 matched donor, double‐unit cord blood transplantation (dCBT) has emerged as a vital alternative for overcoming cell‐dose limitations [1–4]. Notably, while dCBT begins with the infusion of two units, the process typically evolves into single‐unit dominance, making sustained donor–donor mixed chimerism an exceedingly rare clinical phenomenon [5–9].

The clinical management of dCBT is further complicated by high relapse rates, ranging from 20% to 60% [10, 11]. When relapse occurs in a patient with donor–donor chimerism—particularly one lacking a suitable native HLA match—clinicians face a significant dilemma due to the absence of standardized donor selection protocols. In this report, we present a case of relapsed diffuse large B‐cell lymphoma (DLBCL) where persistent donor–donor chimerism offered a unique therapeutic opening. By adopting the HLA genotype of the emergent dominant donor as the template for selection, we successfully performed a second allo‐PBSCT. This strategy achieved complete chimerism and sustained remission for over 4 years, suggesting that a dominant donor’s HLA profile can effectively guide subsequent donor selection when conventional options are unavailable.

## 2. Case Presentation

### 2.1. Initial Diagnosis and First‐Line Treatment

**FIGURE 1:**
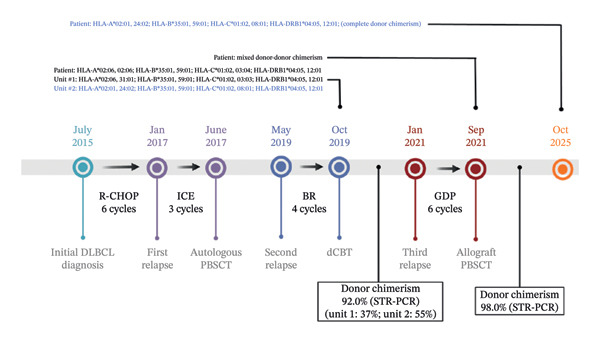
Clinical course and treatment timeline.

In July 2015, a 36‐year‐old patient with no significant medical history was diagnosed with DLBCL, staged as IIIA with an International Prognostic Index (IPI) of 2. She achieved complete remission (CR) following standard R‐CHOP chemotherapy. However, 18 months later, in January 2017, the disease relapsed. The patient subsequently underwent salvage chemotherapy with the ifosfamide, carboplatin, etoposide (ICE) regimen, followed by autologous peripheral blood stem cell transplantation in June 2017 (Figure 1).

### 2.2. Relapse and dCBT

In May 2019, the patient experienced a second relapse. Although partial remission (PR) was achieved following four cycles of bendamustine and rituximab, a definitive allo‐HSCT was deemed necessary. Due to the lack of an HLA‐matched sibling or unrelated donor, she underwent dCBT in October 2019. The patient’s native HLA genotype was *HLA-A*∗02:06/02:06; *HLA-B*∗35:01/59:01; *HLA-C*∗01:02/03:04; *and HLA-DRB1∗*04:05/12:01 (Figure 1). The transplantation utilized two HLA‐mismatched units: Unit 1 (2/8 allele mismatch) and Unit 2 (3/8 allele mismatch), with both units sharing identical HLA‐B and HLA‐DRB1 loci.

The conditioning regimen consisted of cyclophosphamide, fractionated total‐body irradiation, fludarabine, and anti–thymocyte globulin (ATG). Thirty days posttransplantation, Short Tandem Repeat (STR)‐PCR analysis confirmed 92% total donor chimerism, uniquely composed of 37% from Unit 1% and 55% from Unit 2. This persistent donor–donor chimerism reflected the balanced coexistence of both units within the hematopoietic compartment (Figure 1). During the follow‐up period, the patient recovered from two major infectious complications: cytomegalovirus (CMV) reactivation (630,000 IU/mL) on Day 120, which resolved with ganciclovir, and *Pneumocystis jirovecii* pneumonia at six months post‐dCBT, which was successfully treated with trimethoprim–sulfamethoxazole.

### 2.3. Third Relapse and Innovative Donor Selection Strategy

In January 2021, 15 months post‐dCBT, the patient experienced a third relapse. Although salvage chemotherapy with gemcitabine, dexamethasone, and cisplatin (GDP) induced a PR, a second allo‐PBSCT was clinically indicated for long‐term remission. Initial donor searches were conducted based on the patient’s native HLA genotype. However, these efforts yielded no suitable matches.

**FIGURE 2:**
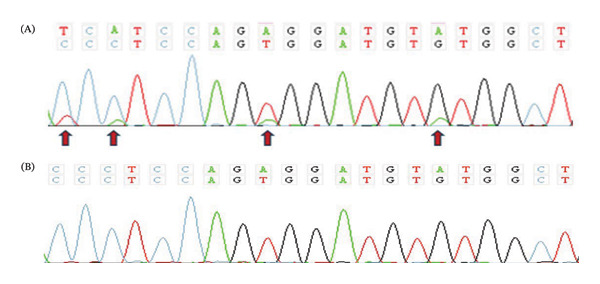
Sequence‐based typing for HLA. (A) High‐resolution sequence‐based typing (SBT) performed prior to the second allogeneic peripheral blood stem cell transplantation (allo‐PBSCT) revealed minor peaks of low signal intensity (red arrows) alongside dominant peaks at the HLA‐A and ‐C loci, indicating persistent mixed chimerism from the initial double‐unit cord blood transplantation (dCBT). (B) Following the allo‐PBSCT, subsequent analysis showed the complete disappearance of the minor peaks, with the sequence data becoming exclusively consistent with the second allo‐PBSCT donor. The confirmed HLA profile was A∗02:01, 24:02; B∗35:01, 59:01; C∗01:02, 08:01; and DRB1∗04:05, 12:01.

Notably, high‐resolution sequence‐based typing (SBT) revealed that the patient’s hematopoiesis was characterized by donor–donor mixed chimerism (Figure 2A). While formal STR‐PCR analysis was not performed at this stage, SBT consistently showed dual peaks at the HLA‐A and ‐C loci, with signal intensities indicating that Unit 2 had become the predominant genotype. Given that the patient’s hematopoiesis had been reconstituted by these specific donor cells, we shifted our search strategy to adopt the Unit 2 HLA profile as the new reference. This approach ultimately led to the successful identification of an HLA‐matched unrelated donor (MUD).

### 2.4. Subsequent Allo‐PBSCT and Long‐Term Follow‐Up

In September 2021, the patient underwent a second allo‐PBSCT from the newly identified MUD following myeloablative conditioning with busulfan, cyclophosphamide, and ATG. Neutrophil and platelet engraftment were achieved on Days 17 and 29, respectively. At the most recent follow‐up in October 2025, more than four years post–second allo‐PBSCT, the patient remains in durable CR with 98% sustained donor chimerism. Notably, the patient’s HLA profile has successfully shifted to match that of Unit 2 (*HLA-A*∗02:01/24:02; *HLA-B*∗35:01/59:01*; HLA-C*∗01:02/08:01*; HLA-DRB1*∗04:05/12:01; Figure 2B). Significantly, the patient has experienced no acute or chronic GVHD and remains free of significant infections.

## 3. Discussion

This case highlights the complex clinical trajectory of a 38‐year‐old patient with triple‐relapsed DLBCL who achieved long‐term remission through a novel transplantation strategy. While cord blood (CB) is a well‐established alternative graft source—offering greater flexibility in HLA matching due to its immunological immaturity—the low progenitor cell dose in single units remains a significant hurdle for adult recipients [2]. This limitation often leads to delayed engraftment and impaired immune reconstitution [3, 4]. To address these challenges, dCBT was utilized to augment the total nucleated cell dose and enhance the likelihood of successful engraftment [5, 12].

In typical dCBT, single‐unit dominance is established within 14–21 days, driven by T‐cell–mediated graft‐versus‐graft alloreactivity [5, 7]. However, this patient exhibited a notable divergence from this standard clinical course. Initial STR‐PCR analysis at Day 30 confirmed stable chimerism, with Unit 1 and Unit 2 accounting for 37% and 55% of hematopoiesis, respectively. Remarkably, even 15 months after the initial dCBT—and amidst a third disease relapse—high‐resolution SBT confirmed the longevity of this state; while Unit 2 had emerged as the predominant fraction, the nondominant unit remained clearly detectable. A defining feature of this case is this exceptionally prolonged donor–donor coexistence. Although limited by the lack of STR analysis immediately preceding the second transplant, our reliance on high‐resolution SBT‐HLA typing at that stage offers compelling evidence that the mixed chimerism—originally detected via STR‐PCR—persisted well beyond the 12‐month threshold typically reported in the literature [5–7, 13]. This phenomenon is likely linked to the unique biological properties of CB, such as the superior proliferative capacity and potent immunomodulatory effects of CB‐derived mesenchymal stromal cells and naïve T‐cells, which may facilitate stable coexistence while mitigating the severity of GVHD [14–17].

The most critical challenge addressed in this report is the management of relapse when no suitable matches exist for a patient’s native HLA profile. In this instance, the emergent dominant donor unit served as a redirected target for donor selection rather than a clinical hurdle. By shifting the search criteria from the patient’s original profile to the emergent dominant HLA genotype (a strategy we term “HLA‐profile shifting”), we successfully identified a MUD for a subsequent allo‐PBSCT. This strategic pivot resulted in robust hematologic recovery and durable CR exceeding 4 years. Beyond dCBT, we propose that this “HLA‐profile shifting” strategy may provide a viable therapeutic pathway for patients relapsing after haploidentical PBSCT, offering a solution for otherwise intractable clinical scenarios.

## 4. Conclusion

This case demonstrates that in the setting of persistent donor–donor mixed chimerism post‐dCBT, the emergent dominant donor genotype can serve as a functional and effective reference for subsequent donor selection. By recognizing this posttransplant shift in the recipient’s HLA landscape, clinicians can effectively expand donor options for rescue transplantation when native HLA matches are unavailable. Our findings suggest that longitudinal chimerism monitoring and HLA retyping in dCBT recipients can provide a viable therapeutic pathway for refractory malignancies, ultimately facilitating long‐term, relapse‐free survival.

## Author Contributions

Youngrok Park designed the report and wrote the manuscript, Seonghan Lee analyzed the data and wrote the manuscript, Jinhang Kim collected the patient’s clinical data, and Jeong‐A Kim provided professional advice and revised the manuscript.

## Funding

No funding was received for this manuscript.

## Disclosure

All authors have read and approved the final version of the manuscript.

## Ethics Statement

The study was approved by the ethics committee of St. Vincent Hospital, College of Medicine, The Catholic University of Korea, Seoul, South Korea.

## Consent

Informed consent was obtained from the patient to publish this report in accordance with the journal’s patient consent policy.

## Conflicts of Interest

The authors declare no conflicts of interest.

## Data Availability

The data that support the findings of this study are available from the corresponding author upon reasonable request.
